# Enhancing the efficiency of coagulation method for sewage treatment by adding sludge

**DOI:** 10.1371/journal.pone.0321286

**Published:** 2025-04-29

**Authors:** Peng Zhao, Lin Li, Xilei Song, Miaokui Wang, Zhengan Zhang, Yuying Li, Yu Zhao, B. Larry Li

**Affiliations:** 1 Henan International Joint Laboratory of Watershed Ecological Security in the Water Source Area of the Middle Route of South-to-North Water Diversion Project, College of Water Resource and Modern Agriculture, Nanyang Normal University, Nanyang, China; 2 Xichuan Ecological Environment Bureau of Nanyang City, Xichuan, China; 3 Academic Affairs Office of Nanyang Normal University, Nanyang, China; 4 College of Civil Engineering and Architecture, Nanyang Normal University, Nanyang, China; 5 Department of Botany and Plant Sciences, University of California, Riverside, California,United States of America.; National Research and Innovation Agency, INDONESIA

## Abstract

The high performance sedimentation tank (HPST) is an efficient water treatment technology, which accelerates the settling rate of flocculates by adding sludge, so as to increase the water treatment load of coagulation sedimentation tank. Its sewage treatment effect is affected by many factors such as sludge dosage, wastewater pH, flocculant dosage, stirring time, settling time, etc. This paper was to study the optimal conditions of HPST, first, some single-factor tests were conducted to preliminarily explore the optimal range of influencing factors, and then response surface methodology (RSM) tests were performed to accurately determine the optimums of significant factors.The results showed that adding sludge can not improve the water quality of coagulation treatment, but it can significantly accelerate the coagulation settlement process, the sludge dosage, the coagulant dosage and sewage pH all impacted significantly on its coagulation effect, and existed inflection points. A model that could guide HPST was obtained by RSM tests. The model optimization and experimental validation showed that the optimal HPST conditions for treating domestic sewage were as follows: the dosage of polyaluminum chloride (PAC) was 1.70 g/L, cationic polyacrylamide (CPAM) dosage was 2.35 mg/L, sewage pH was maintained at 8.0, sludge dosage was 10 mL/L, stirring time lasted for 5 minutes, and settling time lasted for 30 minutes. As a result of these optimized conditions, the turbidity of treated sewage decreased to 1.19 NTU.

## 1. Introduction

Although coagulation is a widely used technology for water treatment [1–3], it has many defects in the traditional coagulation method (TCM). Firstly, the settling rate of the floc is too slow and the settling time is very long, which results in that both the volume and the floor area of the sedimentation tank must be built very large [4,5]; Next, research has demonstrated a significant correlation between the dosage of coagulants and the TCM efficacy.specifically, the optimization of TCM efficiency can be achieved by appropriately adjusting the dosage of coagulants within a specific range. however, this also results in an escalation in water treatment costs [6,7].

In order to solve the defects existing in TCM, heavy media containing fine particulate matter such as sludge, magnetic powder and returned sludge are often used to improve its treatment efficiency [8–10]. The enhancement is attributed to the incorporation of heavy media into the TCM, which promotes the agglomeration of flocs with heavy media particles as their nucleus. Because these flocs have a large specific gravity, they can quickly settle to the bottom of the sedimentation tank. Therefore, this improved method has the advantages of improving the flocculation and precipitation efficiency, improving the water treatment load of the coagulative sedimentation tank and reducing the construction volume and cost [11]. Due to the negative colloidal properties of domestic sewage, it is usually treated with a combination of two coagulants, namely cationic polyacrylamide and polyaluminum chloride [12–14]. The coagulation efficiency is subject to multiple influencing factors such as coagulant dosage, heavy medium dosage and particle size, settling duration, stirring duration, and sewage pH. It is essential to set the values of these factors reasonably for achieving optimal coagulation outcomes [15,16]. Therefore, It is necessary to study the influence of various factors on coagulation effect and its optimum value.

Microsand, magnetic powder and sludge are three commonly employed heavy media for enhancing TCM efficiency. However, compared to sludge, magnetic powder and microsand both exhibit superior improvement effects on TCM; nevertheless, they also possess two evident drawbacks. Firstly, they consume a amount of magnetic powder or microsand, resulting in substantial operating costs. Secondly, separating and recovering magnetic powder or microsand from the sludge increase the complexity of the coagulation process [17,18]. In contrast, utilizing the coagulation-generated sludge as a heavy medium avoids these aforementioned drawbacks. Therefore, in actual engineering treatment, adding sludge as heavy medium is the most commonly used method to improve the efficiency of coagulant water treatment, which is also known as high performance sedimentation tank (HPST).

Single factor experiment is a research method to study the influence of a single variable on the dependent variable.It change only one factor (i.e., the independent variable) in the same experiment, while keeping the other factors (i.e., the control variable) unchanged. This method can clearly reveal the influence trend of a single factor on the experimental results, which is helpful to preliminarily determine the key factors and provide the direction for further research. However, the shortcoming of single-factor experiment is that it neglects the interaction between factors and may not fully reflect the actual situation [19,20]. In order to achieve a more accurate state of experimental results, that is, the gap between the test value of the variable and the optimal value is gradually approaching 0, it is necessary to further improve and optimize the experimental scheme and process, which can be achieved by employing techniques such as orthogonal design or response surface methodology (RSM) [21]. RSM is sequential and can be completed in steps, including screening, factorial, fastest rise and response surface steps [22]. If the variable factor or its value range is not selected properly at the beginning, it can be adjusted during the process [23]. In addition, the precise quadratic regression equation(Eq) can be obtained on the basis of residual bending, which makes the precision of RSM slightly higher than that of orthogonal method in regression. RSM is a powerful statistical method used to optimize the behavior of one or more response variables within a range of multiple input variables. It is widely used in various fields, including chemical engineering, biotechnology, materials science, food science, and industrial engineering [23]. RSM establishes a mathematical model of the relationship between response variables and predictor variables through experimental design, model fitting, and optimization techniques, in order to achieve optimal control of processes or systems [24]. Through the analysis of multiple quadratic regression, the optimal and effective relationship between the target and the influencing factors is established, and the optimal process parameters and the maximum yield are obtained with the least experiments [25]. The Box-Behnken design (BBD) stands out among the many designs of the Response Surface Method (RSM) for its scientific rigor and is widely adopted [26].

At present, there is little research on HPST, and its process parameters are still unclear, which affects its application in engineering practice. Therefore, this study tried to use HPST to treat domestic sewage in order to obtain the influence of each factor on the treatment effect and its optimal value. Firstly, the factors and ranges that affect the treatment effect were obtained by single factor experiment. Then, RSM test is used to determine the optimal value of significant influence factors and achieve the optimal treatment effect.

## 2. Materials and methods

### 2.1 Materials

#### 2.1.1 Domestic sewage.

In this study, the domestic sewage used for coagulation treatment experiment was collected from the septic tank of the student dormitory building in Nanyang Normal University. To reduce water quality fluctuations, water samples were collected at the same time each day, namely about 8:30 every morning. Each collected water sample was used for testing on the same day to reduce the interference of water sample changes on the test results.The turbidity of the raw sewage was 262.1±56.8 NTU, and its COD was 203.2±95.8 mg/L. In order to reduce the influence of water sample concentration difference on the test results, and improve the comparability of different batches of test results, raw sewage water samples with COD of about 200 were selected for experiments, or water samples with very high concentrations were diluted to COD of 200 before being used for treatment tests..

#### 2.1.2 Sludge.

The sludge used in the experiment was derived from the dry sludge of Qingyuan Sewage treatment Plant in Nanyang, it was produced by dewatering the sludge discharged from the secondary sedimentation tank, with a moisture content of less than 70%. The dry sludge was cleaned three times with tap water, and then the cleaned sludge was prepared with distilled water into a mud-water mixture with a moisture content of 92%, which was the heavy medium used to improve the coagulation efficiency.

#### 2.1.3 Coagulants and other materials.

Coagulants used in the test, including CPAM and PAC, were purchased from Gongyi Yiqing Water-Purifying Material Co., Ltd. (Gongyi, China). The coagulants used in the test, including CPAM and PAC, were purchased from Gongyi. The purity of PAC was 30% and the molecular weight of CPAM was 1,200,000.In this study, analytical pure grades of hydrochloric acid (HCl) and sodium hydroxide (NaOH) reagents were used, and homemade deionized water was used to prepare aqueous and standard solutions involved in the study.

### 2.2 Study design

#### 2.2.1 Single factor test design.

A series of gradient flocculation tests were conducted to determine the optimal conditions of factors including sludge addition amount, flocculant dosage, precipitation time, sewage pH, etc., and to study the influence of these factors on HPST treatment efficiency, and to provide data reference for the subsequent RSM test design. The specific experimental design and treatment process are as follows:

The diluted sewage was placed in the container, and the prepared PAC solution was added to it, and stir the sewage solution quickly for a predetermined with ZR4-6 coagulation experimental agitator. Subsequently, the prepared CPAM solution and sludge were added to the sewage, and the sewage solution was stirred slowly for a predetermined time to promote flocculation reaction. Stand the reacted wastewater for a predetermined time to promote floc precipitation.

Gradient test was carried out for each factor to study the influence of the factor change on the effect of coagulation treatment. The turbidity of the sewage after coagulation treatment was detected with the turbidity meter (WZS-186 type turbidity meter produced by Shanghai Yizheng Scientific Instrument Co., LTD.), and the COD was detected by rapid digestion spectrophotometry with the UV-visible intelligent multi-parameter water quality tester (produced by Beijing Lianhua Yongxing Technology Development Co., LTD.). The change of the interface height between supernatant and sludge was observed and recorded in the process of sewage coagulation and settlement. According to the test results, the influence of the change of each factor value on the coagulation treatment effect was analyzed.

In order to clearly study the effect of adding sludge on coagulation, the experiment of TCM treating domestic sewage had also been carried out and compared with HPST, its procedures remained identical to HPST, but no sludge is added.

#### 2.2.2 RSM HPST test design.

The single factor experiment has obvious shortcomings, that is, it ignores the interaction between factors and can not fully reflect the actual situation. Therefore, RSM HPST tests were performed to obtain the best values for each influencing factor. The results of single factor test show that the flocculation process was affected by the sludge dosage, PAC dosage, CPAM dosage, sewage pH and precipitation time. The effect of sedimentation time and sludge dose on flocculation was obvious, and the optimum value was determined by single factor test. However, the factors including CPAM dose, sewage pH, and PAC dose were both positively and negatively correlated with CPAM flocculation efficiency, the best values for these factors obtained through single-factor trials are not necessarily optimal, but close to optimal. Therefore, a Box-Behnken design (BBD) flocculation test was designed and performed to study the optimal value of factors including CPAM dose, sewage pH, and PAC dose. Refer to the results of single factor experiments, the sludge dosage was 10 mL/L, and the stirring and settling time were 5 minutes and 30 minutes. and the amount of CPAM, and the precipitation time was 30 minutes. The source and pre-treatment of sewage used in the test were the same as those used in the single-factor test. The BBD experiments were designed using Design-Expert 13 software, and three levels were set for each factor based on the results of the single-factor test and are shown in Table 1.

**Table 1:** The levels of independent test variables in the experimental study.

Variable code	Variables	Variable levels and corresponding values		
		-1	0	1
**Z**_**1**_	PAC dosage(g/L)	1.35	1.71	2.07
**Z**_**2**_	CPAM dosage(mg/L)	2.3	2.8	3.3
**Z**_**3**_	sewage pH	7	8	9

## 3. Results and discussion

### 3.1 Results and discussion of single factor test

#### 3.1.1 Impact of sludge dosage on HPST treatment effect.

**Fig 1:**
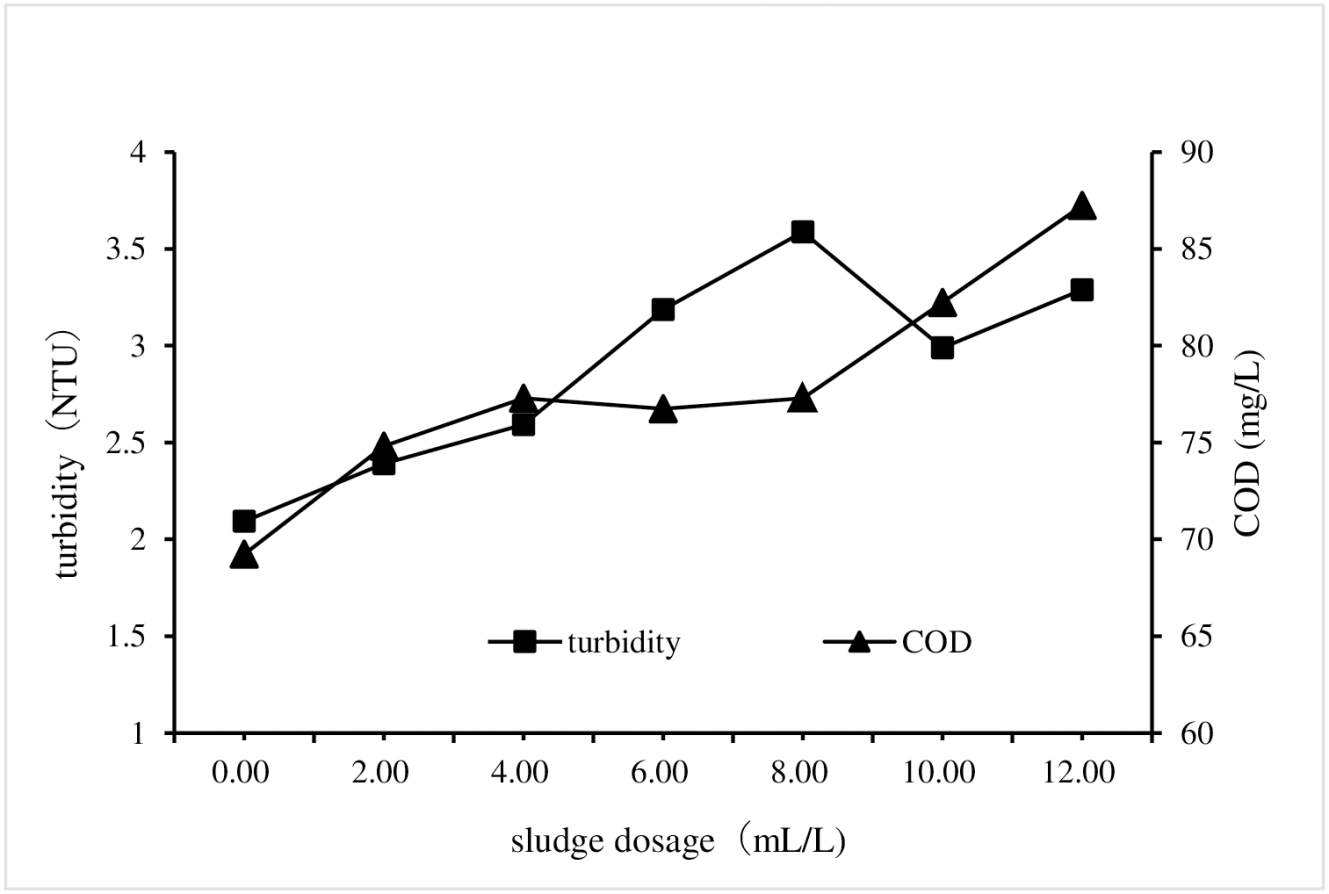
Impact of sludge dosage on HPST.

Change the sludge dosage in HPST test, and maintain the same for other test conditions that the PAC and CPAM dosages were of 1.35 g/L and 1.8 mg/L respectively, and the stirring and settling times were of 5 and 30 mins respectively, and the sewage pH was 7. The turbidity and COD of each treatment test were measured and used to analyze the impact of sludge dosage on HPST treatment effect, and the results were shown in Fig 1.

As illustrated in Fig 1, the turbidity and COD levels exhibit increasing trend with the increment of adding sludge dosage, which suggest that HPST has no obvious advantage over TCM in improving water quality, this could be attributed to the faster settling rate of adding sludge compared to flocs, leading to the disruption of floc structure and generation of smaller, less settleable flocs, ultimately resulting in an elevated concentration of water quality [27]. In addition, the release of organic matter, phosphorus, nitrogen and other substances in the sludge into the sewage may also be an important reason for the deterioration of water quality [28]. Considering cost-effectiveness and the achievement of optimal water treatment results, the subsequent experiments determined that the ideal dosage of sludge is 10 mL/L.

#### 3.1.2 Impact of PAC dosage on HPST.

**Fig 2:**
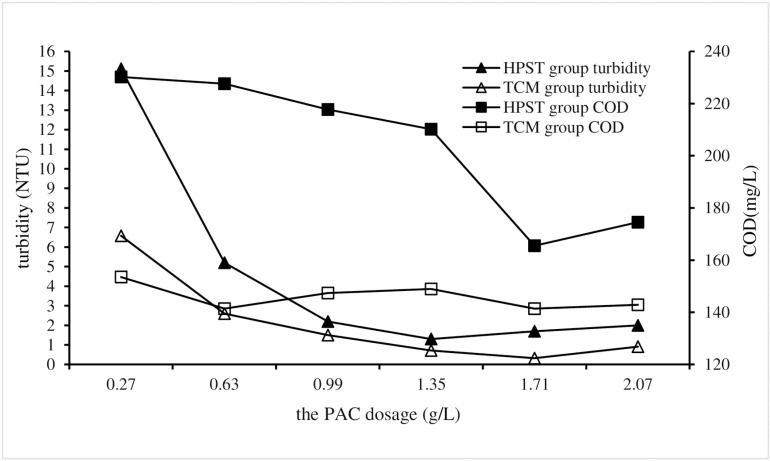
Impact of PAC dosage on HPST.

Change the PAC dosage in HPST test, and maintain the same for other test conditions that the sludge and CPAM dosages were of 10 mL/L and 1.8 mg/L respectively, and the stirring and settling times were of 5 and 30 mins respectively, and the sewage pH was 7. Corresponding TCM tests were also performed as controls against HPST tests.The turbidity and COD of each treatment test were measured and used to analyze the impact of PAC dosage on HPST treatment effect, and the results were shown in Fig 2.

As shown in Fig 2, the turbidity and COD in the experimental group both first decrease to the lowest value with the increase of PAC dosage, and then gradually increase. This phenomenon shows that too much PAC dosage is not conducive to improving the water quality of HPST treatment, and the influences of PAC dosage on the turbidity and COD removals are similar. Through the analysis of the reasons, when the turbidity and COD in the water reach the lowest gradient level, the PAC dosage will become overloaded and react with other substances in the sewage, forming a suspended substance which is difficult to precipitate, and deteriorate the water quality [29,30]. Moreover, according to numerical characterization, the treatment efficiency of TCM group was better than that of HPST group. In addition, according to the gradient treatment levels generated by different doses of PAC, when the added dose of PAC is 1.35g/L, the turbidity of the TCM group and the HPST group is reduced to the lowest value of 0.3 NTU and 1.3 NTU, respectively. When the added dose of PAC is 1.71 g/L, the turbidity of the TCM group and the HPST group is reduced to the lowest value. The COD of the TCM group and the HPST group decreased to the lowest value of 141 mg/L and 165.5 mg/L, respectively. After comprehensive consideration of cost, treatment effect and other factors, the optimal dosage of PAC for HPST treatment process was determined to be 1.71 g/L.

#### 3.1.3 Impact of CPAM dosage on HPST.

**Fig 3:**
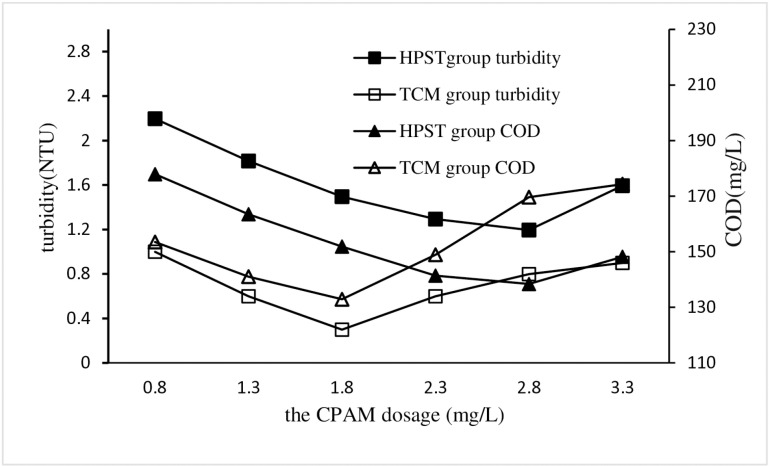
Impact of CPAM dosage on HPST.

Change the CPAM dosage in HPST test, and maintain the same for other test conditions that the sludge and PAC dosages were of 10 mL/L and 1.71 g/L respectively, and the stirring and settling times were of 5 and 30 mins respectively, and the sewage pH was 7. Corresponding TCM tests were also performed as controls against HPST tests.The turbidity and COD of each treatment test were measured and used to analyze the impact of CPAM dosage on HPST treatment effect, and the results were shown in Fig 3.

The HPST test results in Fig 3 shows that with the increase of CPAM dosage, both the turbidity and COD of the sewage exhbited a trend of first decreasing and then increasing. The impact of CPAM on the treatment effect of coagulation water is similar to that of PAC, that is, excessive CPAM is not conducive to the coagulation of sewage, the reason for this phenomenon is that the excess CPAM converts the precipitated floc into positively charged colloids and redissolves in the sewage, which worsens the water quality [31]. Comparing the coagulation efficiencies between HPST and TCM, it is observed that TCM has achieved better water quality treatment effect than HPST.. When the CPAM dosage is 2.8 mg/L, the lowest values of turbidity and COD obtained by HPST are 1.2 NTU and 141.4 mg/L, respectively, while the CPAM dosage of TCM is 1.8 mg/L, and the lowest values of turbidity and COD obtained are 0.8 NTU and 132.9 mg/L, respectively. Considering cost-effectiveness and achieving optimal water treatment results, the optimal CPAM dosage for HPST was determined to be 2.8 mg/L.

#### 3.1.4 Impact of sewage pH on HPST.

**Fig 4:**
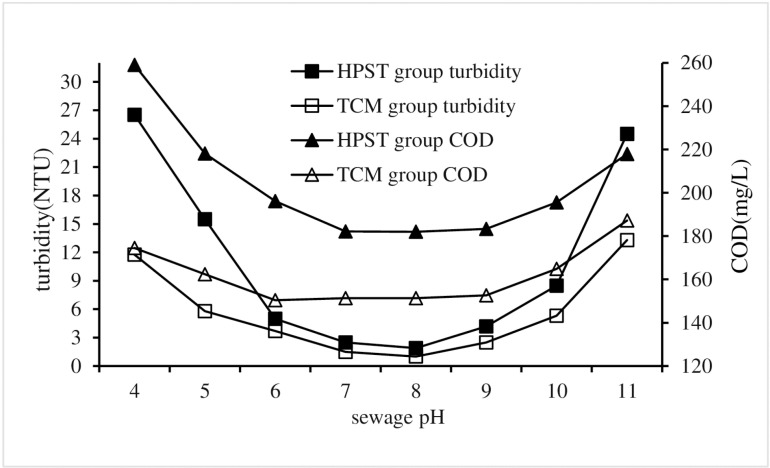
Impact of sewage pH on HPST.

Change the sewage pH in HPST test, and maintain the same for other test conditions that the sludge, CPAM and PAC dosages were of 10 mL/L, 2.8 mg/L and 1.71 g/L respectively, and the stirring and settling times were of 5 and 30 mins respectively. Corresponding TCM tests were also performed as controls against HPST tests.The turbidity and COD of each treatment test were measured and used to analyze the impact of sewage pH on HPST treatment effect, and the results were shown in Fig 4.

Fig 4 shows the COD changes of both HPST and TCM as follows: initially, a decline is observed when the pH falls below 6; subsequently, it remains relatively constant within a pH range of 6–9; finally, an elevation occurs when the pH exceeds 9. The HPST and TCM exhibit minimum COD values of 182.0 and 151.3 mg/L, respectively, accompanied by sewage pH levels of 8 in both cases. These findings indicate that sewage pH exerts a significant influence on coagulation efficiency, with an optimal pH range of 6–9 for achieving effective coagulation. Coagulation performance was observed to be suboptimal when the sewage pH dropped below 6, which can be attributed to that the presence of abundant hydrogen ions in the sewage led to the neutralization of negative charges carried by colloidal particles, which is not conducive to the coagulation and sedimentation of particulate matter [32]. Because of its own positive charge, CPAM repulses positively charged groups in wastewater treatment, which limits the charge neutralization advantage of CPAM. When CPAM is used to treat wastewater, the charge neutralization advantage has a direct effect on the reduction of COD concentration in wastewater [33,34].

The test results also revealed a decline in the coagulant performance when the pH of the sewage exceeded 9. This phenomenon can be attributed to the presence of a large number of hydroxyl ions in sewage, as they effectively neutralized the positive charge of CPAM and diminished the charge neutralization effect of CPAM [35].

The correlation between changes in sewage pH and turbidity closely resembles that observed for COD, suggesting a shared mechanism underlying the pH influence on both turbidity and COD levels. The turbidity of the HPST group was observed to be higher than that of the TCM group under identical treatment conditions. The minimum turbidity values of HPST and TCM were 1.89 and 0.99 NTU, respectively.

#### 3.1.5 Impact of settling time on HPST.

**Fig 5:**
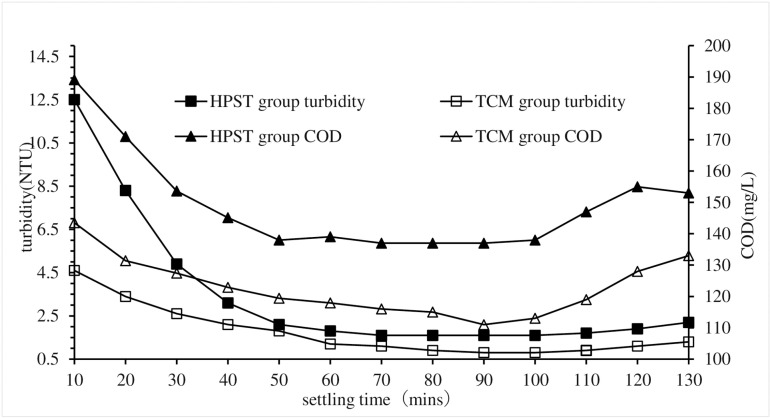
Impact of settling time on HPST.

Change the settling time in HPST test, and maintain the same for other test conditions that the sludge, CPAM and PAC dosages were of 10 mL/L, 2.8 mg/L and 1.71 g/L respectively, and the stirring times was of 5 mins, and the sewage pH was 7. Corresponding TCM tests were also performed as controls against HPST tests.The turbidity and COD of each treatment test were measured and used to analyze the impact of settling time on HPST treatment effect, and the results were shown in Fig 5.

As depicted in Fig 5, the COD and turbidity of both the HPST and TCM groups exhibited an initial rapid decline, followed by stabilization, and finally a gradual increase, which suggests that excessively prolonged sedimentation time is not conducive to effective coagulation. The underlying reason for this phenomenon lies in the presence of a substantial amount of organic matter within the flocs, which undergoes fermentation and gas release, leading to their upward flotation [36,37]. The COD and turbidity of HPST group were always higher than those of TCM group during the whole precipitation process. After approximately 80 minutes of precipitation, the HPST group reached the minimum COD of 137 mg/L and turbidity of 1.6 NTU, while the TCM group took 90 minutes to reach their respective minimum values (COD of 131 mg/L, turbidity of 0.8 NTU). This phenomenon indicates that adding sludge has no obvious promoting effect on the improvement of water quality in the treatment of wastewater by coagulation, but it obviously speeds up the settling rate of floc and shortens the coagulation and precipitation time.When the sedimentation time of HPST exceeded 50 minutes, the decrease in COD and turbidity of sewage were negligible, but it would significantly increase the operating and construction costs of the sedimentation tank. Therefore, the suitable sedimentation time for HPST was determined to be 50 minutes.

#### 3.1.6 Impact of adding sludge on the settling rate of flocs.

In order to study the impact of adding sludge on the settling rate of floc, a set of comparative tests between TCM and HPST were performed.The test conditions for HPST were that the sludge, CPAM and PAC dosages were of 10 mL/L, 2.8 mg/L and 1.71 g/L respectively, and the stirring times was of 5 mins, and the sewage pH was 7. The change of the interface height between supernatant and sludge was observed and recorded in the process of sewage coagulation and settlement.The test conditions of TCM were consistent with those of HPST except that no sludge was added

**Fig 6:**
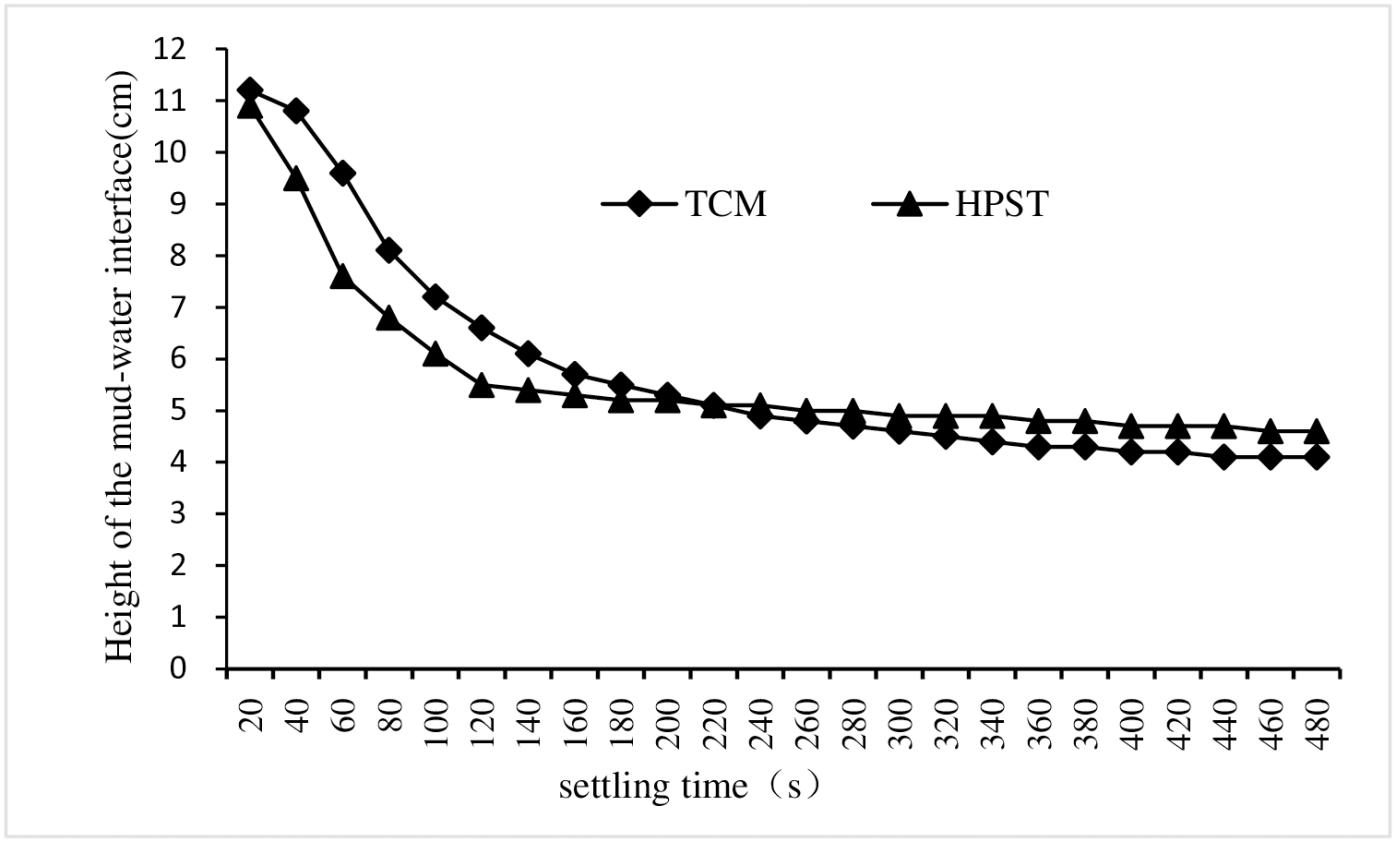
The dynamic change of the interface height between supernatant and sludge.

As depicted in Fig 6, these variations in interface height exhibited a similar trend of rapid decline, followed by stabilization, and ultimately a gradual rise,and the flocculant settling speed of HPST is significantly faster than that of TCM. In addition, the two-phase interface height of HPST group remained stable after 220 seconds of precipitation, while the two-phase interface height of TCM group still showed a downward trend after 480 seconds.The experimental findings demonstrate that the addition of sludge promoted the settling of flocs,which was attributed to the fact that the added sludge help to aggregate particles and form flocs with larger volume and mass, these flocs not only accelerate their settlement but also contribute to improved settling efficiency. Consequently, the addition of sludge can effectively enhance flocculate settling and expedite the coagulation and precipitation process. This ultimately improves the efficiency of sewage treatment in coagulation sedimentation tanks, reduces construction volume, saves costs, and minimizes required area. Consequently, this approach holds promising prospects for practical engineering applications.

### 3.2 Results and discussion of the RSM experiment

#### 3.2.1 RSM experiment results.

According to the BBD test scheme, a total of 17 groups of coagulation tests were conducted, including 12 factorial tests and 5 central tests aimed at inspecting errors.The variable values and the actual turbidity of each coagulation test are presented in Table 2. The results indicate that the turbidities of the 5 central tests were significantly lower compared to the others, which aligns with the findings from the single factor test and demonstrates that while the central point values of the influencing factors in RSM tests were reasonable, they may not necessarily be optimal. Further RSM analysis is required to obtain optimal values [38].

**Table 2:** The actual response values and predicted response values of BDD tests.

Run	the PAC dosage (mg/L)	the CPAM dosage (mg/L)	the sewage pH	The response value of turbidity (NTU)	
				actual	predicted
1	1.71	2.8	8	1.26	1.27
2	2.07	2.8	7	2.74	2.78
3	1.35	2.3	8	2.01	1.97
4	1.35	2.8	9	2.72	2.68
5	1.71	2.8	8	1.34	1.27
6	1.35	3.3	8	1.92	2.04
7	2.07	3.3	8	3.01	3.05
8	1.71	2.8	8	1.34	1.27
9	1.71	2.3	9	1.59	1.67
10	1.71	2.8	8	1.13	1.27
11	1.71	2.3	7	1.73	1.82
12	1.71	3.3	7	1.84	1.76
13	1.35	2.8	7	2.28	2.23
14	2.07	2.8	9	3.18	3.23
15	1.71	2.8	8	1.28	1.27
16	2.07	2.3	8	2.19	2.06
17	1.71	3.3	9	2.89	2.80

#### 3.2.2 Model fitting.

The quadratic equation(Eq) model (Y) was formulated based on linear, quadratic, and cross terms as per Equation (1) in the following manner:


$$Y=A_{0}+A_{1}Z_{1}+A_{2}Z_{2}+A_{3}Z_{3}+A_{12}Z_{12}+A_{13}Z_{13}+A_{23}Z_{23}+A11Z12+A22Z22+A33Z32$$ (1)

The Eq (1) represents the correlation between variables and the corresponding response, the term “Y” in this study refers to the response variable that is being modeled, specifically sewage turbidity(NTU); the variables Z_1_, Z_2_, and Z_3_ represent the first-order terms in this study, namely the PAC dosage (g/L), the CPAM dosage (mg/L), and the pH value of sewage; the terms Z_1_^2^, Z_1_^2^ and Z_2_^2^ represent the quadratic components; and the terms Z_12_, Z_13_ and Z_23_ denote the corresponding interaction effects between two variables, respectively A_0_ was a constant term; A_1_, A_2_ and A_3_ refer to the primary linear coefficients of the PAC dosage (g/L), the CPAM dosage (mg/L) and the sewage pH, respectively; A_11_, A_22_ and A_33_ represent the coefficients of their secondary term, respectively; and A_12_, A_13_ and A_23_ represent the interaction term coefficients among variables, respectively [39].

The response results of the model were analyzed, and analysis of variance (ANOVA) was utilized to evaluate the feasibility of establishing a quadratic equation model between the variables and responses. The statistical significance of the quadratic equation model and test variables was assessed using F tests and p values at a 95% confidence level. The model’s quality was assessed by examining the coefficient of determination R^2^ and adjusted R^2^. Furthermore, the interaction effects of the factors (Z_12_, Z_13_ and Z_23_) on the response variable were analyzed through three-dimensional plots and two-dimensional contour graphs [40].

The regression simulation was performed using the data from Table 3, based on Eq (1), to establish a ternary quadratic polynomial regression model that relates the response to the variables. The resulting equation in terms of actual factors is presented as Eq (2) below.

**Table 3:** ANOVAs for the response surface of Eq (2).

Source		Sum of Squares	Df	Mean Squares	F value	p-value Prob > F	Remark
Model	Eq (2)	7.24	9	0.8042	54.75	< 0.0001	significant
Z_1_-the PAC dosage(g/L)	Eq (2)	0.5995	1	0.5995	40.81	0.0004	
Z_2_-the CPAM dosage(mg/L)	Eq (2)	0.5724	1	0.5724	38.97	0.0004	
Z_3_-the sewage pH	Eq (2)	0.4005	1	0.4005	27.27	0.0012	
Z_12_	Eq (2)	0.207	1	0.207	14.09	0.0071	
Z_13_	Eq (2)	0	1	0	0	1	
Z_23_	Eq (2)	0.354	1	0.354	24.1	0.0017	
Z_1_^2^	Eq (2)	3.15	1	3.15	214.47	< 0.0001	
Z_2_^2^	Eq (2)	0.0916	1	0.0916	6.24	0.0412	
Z_3_^2^	Eq (2)	1.49	1	1.49	101.48	< 0.0001	
Residual	Eq (2)	0.1028	7	0.0147			
Lack of Fit	Eq (2)	0.0732	3	0.0244	3.3	0.1396	not significant
Pure Error	Eq (2)	0.0296	4	0.0074			
Cor Total	Eq (2)	7.34	16				
R^2^_Pre_	Eq (2)	0.8341					
R^2^_adj_	Eq (2)	0.9680					


$$Y=78.283-25.605Z_{1}-9.69Z_{2}-10.962Z_{3}+1.264Z_{12}+0.595Z_{23}+6.674Z_{1}2+0.59Z_{2}2+0.595Z_{3}2$$ (2)

The analysis of variance (ANOVA) was performed for the quadratic model of the response surface, as represented by Eq (2). Subsequently, a significance test was conducted to assess the impact of each variable, and the corresponding results are presented in Table 3. The significance of the model terms can be inferred when the “p values Prob > F” are less than 0.0500[41]. In this case, Z_1_, Z_2_, Z_3_, Z_23_, Z_23_, Z_12_, Z_22_ and Z_32_ were all significant model terms, and had significant impacts on sewage turbidity. The model terms Z_1_, Z_2_, Z_3_, Z_23_, Z_23_, Z_12_, Z_22_ and Z_32_ all exhibited significant effects on sewage turbidity in this study. The “p values Prob > F” of the model exhibited statistical significance, as their values were below the threshold of 0.0500. The model terms Z_1_, Z_2_, Z_3_, Z_23_, Z_23_, Z_12_, Z_22_ and Z_32_ exhibited significant impacts on sewage turbidity in this study. The Lack of Fit F-value of 3.30 implies the Lack of Fit is not significant relative to the pure error. The “Pred R-Squared” value of 0.8341 demonstrates a reasonable agreement with the “Adj R-Squared” value of 0.9680, indicating a good fit for Eq (2) and its suitability for predicting HPST turbidity. Table 3 presents the predicted turbidity values for all HPST tests based on Eq (2).

#### 3.2.3 Response surface analysis.

**Fig 7:**
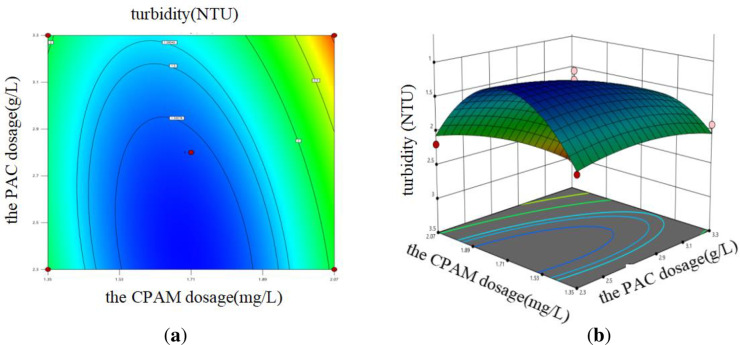
The impact of the interaction between PAC and CPAM dosage on sewage turbidity. (a) Contour diagram; (b) 3D surface diagram.

**Fig 8:**
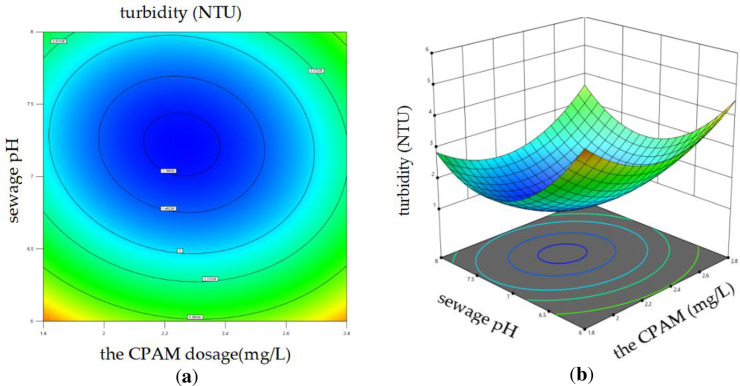
Impact of the interaction between sewage pH and CPAM dosage on sewage turbidity. (a) Contour diagram; (b) 3D surface diagram.

The Eq (2) was employed to conduct ANOVA analysis in order to assess the influence of variable interactions on the coagulation effect. The results revealed that both the interaction between PAC dosage and CPAM dosage, as well as the interaction between sewage pH and CPAM dosage, significantly impacted sewage turbidity. Design Expert 13 software was used to draw the response surface diagrams,

The Eq (2) was employed to conduct ANOVA analysis in order to assess the influence of variable interactions on the coagulation effect. It was found that the combined effect of PAC dose, pH value, and CPAM dose significantly affected the turbidity of sewage. Design Expert 13 software was used to draw the response surface diagrams, as shown in Figs 7 and 8. The steepness of the three-dimensional response surface can be utilized to assess the impact of each factor on sewage turbidity. If the contour graph was oval, it indicated that the interaction effect of the corresponding factors had a significant influence on the sewage turbidity; however, when the contour tended to be circular, the influence was small [42]. The fixed factor was kept constant while investigating the effects of the remaining two factors on the response variable. The data presented in Fig 7 demonstrate that, under constant pH conditions, the turbidity of sewage initially decreases and then increases with increasing doses of both PAC and CPAM. Notably, when the PAC dose ranges from 2.7 to 2.9 g/L and the CPAM dose ranges from 1.53 to 1.89 mg/L, the sewage turbidity reaches its minimum value. Similarly, Fig 8 illustrates that with an increase in both the pH value and CPAM dose of the sewage, while maintaining a constant PAC dose, there is an initial rise followed by a subsequent decline in turbidity change. The minimum turbidity values were observed within the pH range of 7 to 7.5 and CPAM dosage range of 2.2 to 2.4 mg/L.

#### 3.2.4 Optimal coagulation conditions and model validation.

The first partial derivative of Eq (2) is set to zero to solve, and the optimal solidification conditions are obtained as follows: a dosage of 1.70 g/L for polyaluminum chloride (PAC), a dosage of 2.35 mg/L for cationic polyacrylamide (CPAM), and a wastewater pH value of 8.0. The predicted turbidity value of the wastewater under these coagulate conditions is 1.15 NTU. To validate the reliability of the prediction model, two additional experimental runs were conducted using the coagulation conditions obtained from model optimization, These experiments also incorporated sludge as a heavy medium with a dosage of 10 mL, while the stirring time and settling time were set at 5 mins and 30 mins respectively. The experimental results are presented in Table 4. The measured average turbidity of 1.19 NTU closely aligns with the predicted value of 1.15 NTU, demonstrating a minimal difference of only 3.5%, which indicates that the prediction model could serve as a reliable guide for quartz sand-enhanced coagulation.

**Table 4:** Measured and predicted values of sewage turbidity.

coagulation conditions						sewage turbidity (NTU)	
PAC dosage (g/L)	CPAM dosage (mg/L)	Sewage pH	sludge dosage (mL)	Stirring time (minutes)	Settling time (minutes)	Average of measured value	Predicted value
1.70	2.35	8.0	10	5	30	1.19	1.15

## 4. Conclusions

To investigate the optimal coagulation conditions of HPST, initial single-factor tests were conducted to preliminarily explore the optimal range of influential factors, followed by subsequent RSM tests to accurately determine the optimum levels of these factors.The results of the single-factor test indicated that adding sludge does not enhance the efficacy of coagulation treatment in improving water quality, but can speed up the flocculation and precipitation process, and increase the sewage treatment load of coagulation sedimentation tank. The PAC dosage, CPAM dosage and sewage pH all have significant effects on the effect of HPST and show the optimal level. A HPST-guiding model was derived through RSM tests, and the model optimization revealed the optimal conditions for treating domestic sewage as follows:the PAC dosage, CPAM dosage, sewage pH, sludge dosage, stirring time and settling time were 1.70 g/L, 2.35 mg/L, 8.0, 10mL/L, 5 minutes and 30 minutes respectively. Under this condition, the turbidity of the treated sewage is reduced to a minimum of 1.19 NTU.
